# Evaluation of the Di(2-ethylhexyl)phthalate released from polyvinyl chloride medical devices that contact blood

**DOI:** 10.1186/2193-1801-3-58

**Published:** 2014-01-29

**Authors:** Hongyu Luo, Guangyu Sun, Yanping shi, Yong Shen, Kai Xu

**Affiliations:** CFDA Jinan Quality Inspection Center for Medical Devices, 99 Tianluo Road H-T Industrial Development Zone, Jinan, 250000 China; Shandong Hengxin Inspection Technology Development Center, 99 Tianluo Road H-T Industrial Development Zone, Jinan, 250000 China; Shandong Provincial Key Laboratory of Biological Evaluation of Medical Device, 99 Tianluo Road H-T Industrial Development Zone, Jinan, 250000 China

**Keywords:** Di(2-ethylhexyl) phthalate (DEHP), Medical devices, Ethanol/water mixture, Polyvinyl chloride (PVC), Gas chromatography/mass spectrometry (GC-MS), Blood contact

## Abstract

Extraction methods that simulate those used in the clinic are recommended for obtaining extraction solutions. For polyvinyl chloride (PVC) medical devices that have contact with human blood, an alternative medium (ethanol/water mixture) is suggested as an extraction screening vehicle to evaluate the di(2-ethylhexyl)phthalate (DEHP) released. A test comparing the extraction ability between the alternative medium and whole blood from three healthy volunteers has been conducted. An experimental method is provided outlining the chemical analysis of the DEHP released from medical devices made with polyvinyl chloride (PVC). Gas chromatography-mass spectrometry (GC-MS) in the selective ion monitoring (SIM) mode was used to analyze and quantify the extracted DEHP. The linear range of the SIM method was 0.1-200 μg/mL, and the recoveries were 89.6-101.5% and 91.0-98.9% when using the ethanol/water mixture and whole blood as the extraction media, respectively. The validated method demonstrates that it is suitable for the determination of the DEHP released from PVC medical devices that have contact with blood. The results from the determination of the DEHP released will be compared with the limits derived from toxicological data for the parenteral exposure route and certain population groups, and the results will be used in the risk assessment of medical devices.

## Introduction

Patients undergoing medical procedures, such as intravascular therapy, parenteral nutritional support, blood transfusions, hemodialysis, cardiopulmonary bypass (CPB) and extracorporeal membrane oxygenation (ECMO), can be exposed to di-(2-ethylhexyl)phthalate (DEHP), a compound used as a plasticizer in polyvinyl chloride (PVC) medical devices. DEHP has been shown to produce a wide range of adverse effects in experimental animals, most notably liver toxicity and testicular atrophy. Although the toxic and carcinogenic effects of DEHP have been well established in experimental animals, the ability of this compound to produce adverse effects in humans is controversial. As a result, the ability of DEHP and other phthalate esters to produce adverse effects in humans has been a topic of active discussion and debate in the scientific and regulatory communities (U.S.FDA 2001). Since 2001, several countries have initiated safety assessments of DEHP-plasticized PVC medical devices (U.S.FDA 2001; European Commission, 2008; Health Canada 2001; BfArM Germany 2004). In a U S Food and Drug Administration (FDA) report, the tolerable intake (TI) values for two DEHP exposure routes were derived from the available toxicity data. The TI values for the parenteral and oral routes are 0.60 mg/kg/day and 0.04 mg/kg/day, respectively (U.S.FDA 2001).

Over the past few decades, there have been various DEHP-plasticized PVC medical devices used in clinics, such as blood bags, infusion or transfusion tubings, enteral and parenteral nutritional tubings, cardiopulmonary bypass tubings, hemodialysis systems, various tube systems for blood cell separation machines, heat lung packs and leukocyte-reducing filter sets. Of the most widely used devices, nearly half come into contact with human blood or blood components.

For the purpose of assessing the safety of the DEHP released from medical devices during clinical use, an extraction method simulating clinical use would normally be used. In a study focusing on donor exposure to DEHP during plateletpheresis, the blood of 36 healthy donors undergoing plateletpheresis with either continuous or discontinuous apheresis devices was collected, and the serum concentrations of DEHP were determined (Buchta et al. 2003). In two other studies, DEHP exposure was assessed in 11 patients and 21 patients with chronic renal failure undergoing maintenance hemodialysis (Dine et al. 2000; Faouzi et al. 1999). Other studies that have been conducted include the evaluation of the DEHP exposure of voluntary plasma and platelet donors (Koch et al. 2005a; Koch et al. 2005b). Studies on the types of medical devices that have contact with blood usually need to recruit volunteers or study blood collected from patients. However, with such a large variety of devices being used during clinical therapeutic procedures, it is very difficult or impractical to conduct the study in patients or volunteers when a certain type of exposure assessment is needed and an alternative contact medium is preferred. This is especially true when the medical device is still in pre-market evaluation stages and cannot be used on human beings. In a risk assessment study of the DEHP released from PVC blood circuitry during hemodialysis and pump-oxygenation therapy, fresh bovine blood containing heparin (10,000 U/L) was used as the contact medium instead of human blood (Haishima et al. 2004), and it was a good and reasonable choice. Even so, it may still not be very convenient to perform these studies in a laboratory. In this study, an ethanol/water mixture with a density from 0.9373 to 0.9378 g/ml is recommended as a blood alternative, and the mixture is also available as an alternative extraction medium in accordance with ISO3826-1 (ISO 3826-1 2003). This mixture has also been adopted by the European Pharmacopoeia as the extraction vehicle for the evaluation of the DEHP extracted from blood bags (European Pharmacopoeia 7.0). However, when reviewing the test method in both of the documents above, it may be considered an accelerated test method. Briefly, this alternative solvent was used to fill up a blood bag to half the amount of its nominal capacity, and after releasing the air from the bag and incubating it at 37 ± 1°C for 60 ± 1 min, a UV/VIS spectrophotometer was utilized at 272 nm to determine the amount of DEHP. Thus, in addition to using the alternative solution, the test method neither simulated the practical use of the device nor the specific for analysis in most cases. For example, the blood bag was normally manufactured without the use of any adhesives, but many of the tubes would use, which might be an interfering factor when using UV/VIS spectrophotometer. In this study, blood has been collected from 3 healthy volunteers to compare the DEHP extracting abilities of the alternative solution and human whole blood.

There are various analytical methods that can be used to quantitatively determinate the extracted DEHP, including GC, HPLC, GC-MS, LC-MS and even a UV spectrophotometric method (ISO 3826-1 2003; Haishima et al. 2005; Inoue et al. 2005; Han et al. 2005; Ito et al. 2005; Chiellini et al. 2011). Due to the variety of medical devices made with PVC on the market as well as the various intended clinical uses, there is no universal evaluation method for all intended purposes of medical devices made with PVC. In this study, a GC-MS method was used for determination of the released DEHP. The validation of the method included evaluating the precision, accuracy, linearity, sensitivity and selectivity (ICH Q2R1 2005).

The goal of the present study is to recommend and validate an alternative extraction solution for medical devices that have contact with human blood or blood components to use as a screening test for the evaluation of the DEHP released from PVC products. The GC-MS method was established and validation of the methodology was conducted for both the alternative (ethanol/water mixture) and whole blood extractions. The results of the DEHP determinations were then compared with the DEHP tolerable exposure limits as part of the risk assessment of the products.

### Experimental

#### Chemicals and reagents

The DEHP standard was purchased from AccuStandard (125 Market St. New Haven, CT 06513, USA). Ethanol, which was used as part of the extraction solution, was liquid chromatographic grade, was screened to determine the DEHP background and was purchased from Merck KGaA (Darmstadt, Germany). n-Hexane of a grade suitable for liquid chromatography was used as the solvent of the working standard and as a dissolving regent during the sample preparation procedure. It had been screened for its DEHP background and was purchased from Merck KGaA (Darmstadt, Germany). In addition, the disodium salt of EDTA and heparin sodium salt, which were used in this paper as anticoagulants when collecting blood, were purchased from the Xilong Chemical Factory (Guangdong, China) and Sigma (3050 Spruce Street, St Louis, USA), respectively. The pure water used as part of the extraction solution was prepared using an ULTRAPURE purification system (Shanghai, China) at a resistance greater than 18 Ω.

### Preparation of the working standards

Approximately 20 mg of the DEHP standard was accurately weighed out and diluted with n-hexane to 10 mL in a volumetric flask. This solution was labeled the 2000 μg/mL stock standard and was stored in the fridge. The solution is stable for 1 month. According to the released DEHP level in the sample solution or its predicted level, a proper working standard range within the linear range of 0.1 μg/mL-200 μg/mL was selected. The stock standard was diluted stepwise with n-hexane to prepare at least 5 concentrations of working standards. For the whole blood sample, a spiked standard method was used because of its relatively complicated matrix.

### Sample preparation

#### General

In principle, the method for DEHP extraction from PVC medical devices usually takes into account the intended clinical use of the medical device. For example, in medical devices contacting blood, such as a dialyzer, oxygenator, or blood circuit, an appropriate amount of anticoagulant, such as a heparin salt or an ethylenediamine-tetraacetic acid (EDTA) salt, is added into the blood and circulated into the system under the same conditions as those for clinical use (circulation time, circulation rate and temperature), and a suitable amount of the circulated blood is collected as the test sample. In addition, an ethanol/water mixture with a density from 0.9373 to 0.9378 g/mL is suggested as an alternative solution in this paper for a screening test to evaluate the DEHP released from devices that contact blood.

### Extraction with ethanol/water

It may not be very practical or convenient to use blood or its components as the extraction media to evaluate the DEHP released from medical devices because in many cases, it is difficult to collect enough blood or to recruit enough volunteers for the study. Especially for products in a preclinical stage, it is almost impossible to collect body fluids from patients, and a simulated method may be preferred. Even for a post-market product intended to perform this determination, it may be not convenient to recruit volunteers or collect samples from patients. In these situations, the use of an alternative medium is recommended. Therefore, using an ethanol/water mixture with a density from 0.9373 to 0.9378 g/mL as an alternative extraction vehicle instead of blood and following the procedures described in the General section or other procedures has been justified. The density of the ethanol/water mixture, which ranged from 0.9373 g/ml to 0.9378 g/ml (20°C), was determined with a pycnometer. (ISO 3826-1 2003).

A certain amount of the extracted sample was taken and dried at 50°C under vacuum in a vacuum drying oven to complete dryness. The sample was equilibrated to room temperature and an equal volume of n-hexane was added and followed by vortexing the solution vigorously for 1 min. This constituted the sample solution. If the result of the determination of DEHP was acceptable compared with the limit of tolerable exposure for human beings, no further tests were needed on the blood.

### Extraction with blood

If the released DEHP determination result with the alternative media is not acceptable, an extraction with blood may be used.

The extraction solution was prepared using the method described in the General section above or another method that has been justified with blood containing anticoagulants (EDTA salts or heparin, etc). Then, 0.5 mL of extraction solution was transferred into centrifuge tubes, 2 mL of n-hexane was added, and the solution was vortexed for 1 min. It was then centrifuged for 10 min at 3000 rpm, and the supernatant (n-hexane layer) was considered the sample solution.

### GC-MS conditions

**Figure 1:**
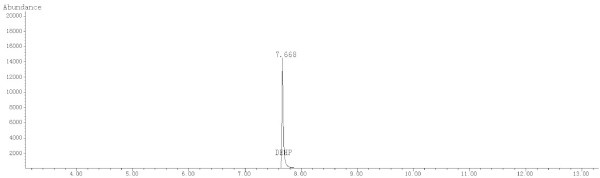
**Chromatogram of the DEHP standard solution (10 μg/mL) in the SIM Mode.**

**Figure 2:**
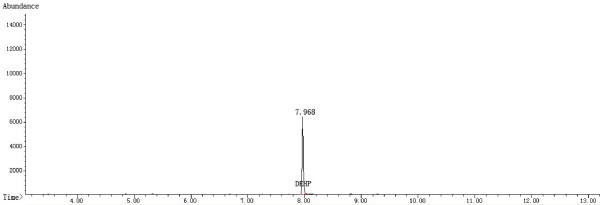
**Chromatogram of the sample solution (ethanol/water mixture) in the SIM Mode.**

**Figure 3:**
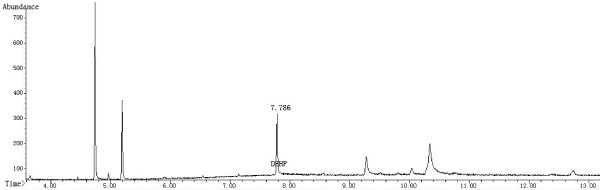
**Chromatogram of the sample solution (blood) in the SIM Mode.**

**Figure 4:**
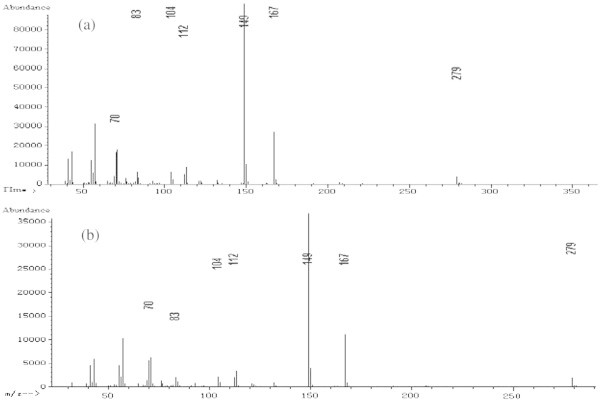
**MS Spectrum of the DEHP standard and the ethanol/water mixture sample solution.** [Note **(a)** for standard and **(b)** for sample solution].

GC-MS was conducted using an Agilent 7890 GC and an Agilent 5975C mass spectrometer equipped with an electron ionization (EI) source and a quadrupole analyzer. The GC capillary column was an HP-5MS (30 m × 0.25 mm × 0.25 μm, Agilent J & W GC columns, USA). The fragment ion with a *m/z* = 149 was selected for the GC-MS SIM detection. The ion source and inlet temperatures were 230°C and 280°C, respectively. In addition, the quadrupole and auxiliary temperatures were 150°C and 280°C, respectively. A split ratio of 5:1 was used, and a mass scan range from 45-450 amu was employed. The EI energy was 70 eV. The starting column temperature was 150°C, which was held for 0.5 min, and the temperature was then raised to 280°C at 20°C/min, where it was held for 7 min. In addition, the gas flow was set at 1.5 mL/min, and the solvent delay time was 3.5 min. Figures 1, 2, 3 and 4 show the chromatograms and MS spectra obtained under these conditions.

## Results and discussion

### DEHP background

Phthalates began to be used in the plastics industry more than 80 years ago. DEHP is added to PVC material that is used in food packaging material, medical products, plastic toys, vinyl upholstery, shower curtains, adhesives, and coatings (Fernández et al. 2012). Various phthalate esters have been reported to be present in the environment, including in outdoor air, water, soil, indoor air and dust, sediment, seafood and human tissue (Fernández et al. 2012; Zeng et al. 2011; Santhi and Mustafa 2013). As a result, during the test procedure, reducing the DEHP background that may result from reagents, utensils and instrument systems to levels as low as possible is necessary. In this study, all of the reagents, especially the n-hexane used in standard and sample preparation, were subjected to DEHP background screening to ensure that the target chemical was at the lowest level possible. The same screening was applied to ethanol. In some cases, there were very different levels of DEHP background in the screened regents even though all of them are labeled as chromatography grade.

**Figure 5:**
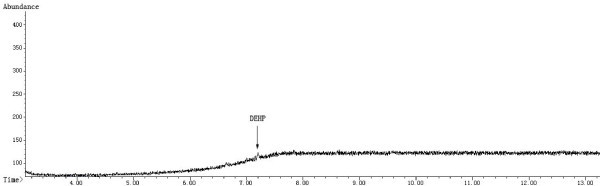
**Chromatogram of the n-hexane regent screened in the SIM Mode.**

Another important factor that can contribute to background DEHP is the utensils; in this study, all of the utensils used were made of glass to avoid DEHP being released from plastic utensils. Pretreatment of the utensils to reduce any DEHP residue on them was performed. Usually utensils are heated to high temperatures, such as to 250°C for more than 10 h or to 400°C for 2 h (Haishima et al. 2005; Loff et al. 2004). However, in this study, some of the utensils, such as transfer pipettes and volumetric flasks, are used for accurately transferring solutions or to prepare the standard solutions, and it is recommended suggested that they are not heated to high temperatures after calibration. Therefore, these types of glass utensils were rinsed with n-hexane that was previously screened for background DEHP (see Figure 5) and were individually validated using the previously described GC-MS conditions to ensure the DEHP background was as low as possible.

### Comparison between the extraction abilities of the water/ethanol mixture and blood

A considerable number of medical devices made from PVC material come into contact with blood or blood components during use. Although there is much literature regarding methods for determination of DEHP in whole blood, plasma or serum, it is not very practical to use blood or its components as extraction media to evaluate the DEHP released from medical devices everytime the equipment is used.

**Figure 6:**
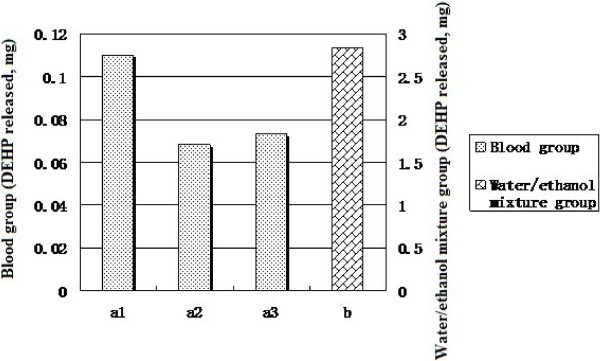
**Comparison of the extractions with blood and the water/ethanol mixture.**

In this study, blood samples from three healthy volunteers were collected, anticoagulated with an EDTA salt and stored in glass containers. This lot of blood samples was used to simulate the practical use of the devices as the first group; in the second group, a water/ethanol mixture with a density (ρ) of 0.9378 g/ml (20°C) was utilized to extract the DEHP under the same conditions (the same extraction media volume, the same dripping rate and the same ambient temperature) as those for the first group. The results of this determination are provided in Table 1 and Figure 6.

**Table 1:** **Comparison of the extractions between blood and the water/ethanol mixture**

***Blood group (DEHP released, mg)***			***Water/ethanol mixture group (DEHP released, mg)***
1^a^	2^a^	3^a^	
0.110	0.068	0.073	2.84

^a^The numbers 1, 2, and 3 correspond to whole blood samples from three volunteers.

While a small sample size was used and only one type of medical device was tested, the outcomes based on the scenario above are quite different when comparing the blood samples with the water/ethanol mixture. Apparently, the extraction ability of the water/ethanol mixture is much higher than that of blood. In addition, the effect of using blood with different properties has not been taken into consideration. For example, blood with high cholesterol and high triglyceride contents might be able to extract more DEHP than blood from healthy people. A study on the correlation between an increase in serum DEHP concentration and the endogenous serum lipid concentration has indicated a weak association between the concentration of triglycerides in the serum and the relative increase in the serum DEHP concentration. In contrast, no correlation was found between an increase in the DEHP concentration and the concentration of cholesterol in serum (Buchta et al. 2003). As a result, when blood is not available, the use of an alternative medium is preferred, and if the result of the DEHP determination is acceptable, then no further tests need to be performed using blood.

### Methodology validation

#### Repeatability and accuracy

The linear range of the determination of DEHP using this study is 0.1-200 μg/mL when using n-hexane as the solvent. The correlation coefficient is greater than 0.999.

To validate the repeatability, standard solutions with four different concentrations have been used to represent high, intermediate, low, and very low concentrations within the linear range of 0.1-200 μg/mL, and 6 replicates were used at each concentration. The results are listed in Table 2.

**Table 2:** **Repeatability validation results**

***Concentration (μg/mL)***	***Mean (Peak area)***	***Standard deviation (SD)***	***Relative standard deviation (RSD%)***
Very low (0.1)^a^	7693	299	3.88
Low (1.0)	215724	3347	1.55
Intermediate (50.1)	14429286	502984	3.48
High (200.6)	48864521	1841486	3.76

^a^This concentration was tested at a different time and did not exhibit linearity with the other concentrations.

**Figure 7:**
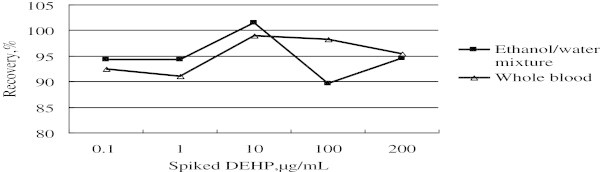
**Results of the recovery validation.**

In addition, a recovery test was used to evaluate the accuracy of the method and was conducted by spiking samples at 5 different concentrations levels using 3 replicates at each level. The results are listed in Table 3 and Figure 7.

**Table 3:** **Recovery validation results**

***Spiked concentration μg/mL***	***Spiked sample recovery***			
	Spiked in ethanol/water (n = 3)		Spiked in blood (n = 3)	
	Mean recovery (%)	RSD (%)	Mean recovery (%)	RSD (%)
0.1	94.23	2.33	92.43	3.88
1	94.23	4.51	91.04	6.71
10	101.53	8.80	98.90	0.57
100	89.58	7.16	98.15	2.24
180, 200^a^	94.60	5.02	95.35	7.86

^a^180 μg/mL and 200 μg/mL were spiked for the ethanol/water test and blood test, respectively.

### Intermediate precision

The experiment was performed using the same method, instrument, and device lots (transfusion sets were used), but on different days by different analysts. Each group prepared 6 sample replicates (the water/ethanol mixture was used as the extraction medium). The results are listed in Table 4.

**Table 4:** **Intermediate precision test results**

***Group***	***Mean (n = 6) μg/mL***	***Total RSD (%) within the two groups (n = 12)***
Group 1	13.17	9.00
Group 2	12.66	

### Detection limit (DL) and Quantitation limit (QL)

As has been discussed in section DEHP background of this paper, normally there is always DEHP background present during the testing procedure because of various contamination sources, and as a result, lowering the background DEHP would be a primary goal before sample testing, especially for low concentration level tests. Furthermore, the DEHP background level plays an important role when determining the DL and QL values. When using n-hexane, which has been screened and found to contain almost no background DEHP (Figure 5), to prepare a series of very low level DEHP standards, after injecting them into the instrument and using the method based on using signal to noise ratios of approximately 3:1 and 10:1 to assess the DL and QL, the DL and QL of the method in the SIM mode were approximately 1 ng/mL and 2.5 ng /mL, respectively. However, in most cases, background DEHP would present, although it may be at a very low level and in this study basically comes from the ethanol/water mixture and the blood itself. In this case, the DL and QL value determinations usually refer to ICH Q2 (R1) 6.3 and 7.3 and use the calculation formulas as follow, respectively:

where σ = the standard deviation of the response and S = the slope of the calibration curve.

The σ values were obtained in this study by injecting blank samples (ethanol/water mixture and whole blood) and calculating the standard deviation of these responses using 10 replicates of each. The calculated DL and QL values in this study were 3.08 ng/mL and 9.35 ng/mL for blood and 2.91 ng/mL and 8.81 ng/mL for the ethanol/water mixture, respectively. It should be noted that just as described in section DEHP background, the screening test results within the same regent groups indicated that there might be different DEHP background levels present; therefore, sometimes it may be necessary to report the DEHP DL and QL case by case when the regent supplier or even the lot number is changed.

### Specificity

It has been verified that the analytical procedure is specific for the determination of DEHP and that the solvents, extraction media, including the water/ethanol solution, blood components, and blood anticoagulants, including both the EDTA salt and heparins, do not interfere with the DEHP.

### Sample determination results

The DEHP from the three different types of medical devices listed was determined using the established GC-MS method described. The devices were single use transfusion sets, single use leukocyte-reducing filters and tube systems for haemodialysis. The exposure route to human beings for all of these devices is the parenteral route. The sample preparation methods were conducted simulating the clinical use of these devices. The test results are listed in Table 5.

**Table 5:** **Sample determination results**

***Medical devices***	***Extraction media***	***DEHP released, mg***
Transfusion set for single use^a^	Water/ethanol mixture	2.84
	blood	0.068-0.073^b^
Leukcyte-reducing filter for single use	Water/ethanol mixture	1.2
Tube system for haemodialysis use	Water/ethanol mixture	11.6

^a^100 mL of the extraction media (both the water/ethanol mixture and blood) was used.

^b^Blood from three healthy volunteers

According to the FDA DEHP safety assessment document, the tolerable intake (TI) values for DEHP are 0.6 mg/kg(body mass)/day for the parenteral route, and in the absence of specific information, body masses of 70 kg for adults, 10 kg for children, and 3.5 kg for neonates should be used (ISO 10993-17 2002). Using the TI value of 0.6 mg/kg(body mass)/day, the calculated DEHP tolerable exposures (TEs) for human beings are 42 mg/day for adults, 6 mg for children and 2.1 mg for neonates, respectively. Apparently, all of the determination results are less than the TE value for adults, which means that the DEHP released from these medical devices is safe for adults. For the transfusion set, the result from the alternative media is more than the TE value for neonates, but when blood was used, which will be the practical contact media during clinical use, the released DEHP was much less than the TE value for neonates. This result indicates that the amount of DEHP released from this product is also safe, even for neonates. When reviewing the tube system for haemodialisis use, the result is much higher than the TE values for children and neonates; therefore, a further extraction with blood may be needed if the product is intended for these types of populations. If those results can still not conform to the TE values of these two groups, then a risk analysis should be conducted or the selection of an alternative product is recommended. However, it should be noted that with more and more concern with the toxicity of DEHP during the past ten years, especially the increased risk in children (U.S.FDA 2001), many products have been developed using an alternative plasticizer or an alternative material for children and neonates.

## Conclusion

A useful and convenient alternative solvent for human blood is recommended for use in a screening test to evaluate the DEHP released from medical devices that have contact with blood. A GC-MS method has been established for the determination of the amount of DEHP in both the alternate extraction solution and whole blood. This method is feasible for analyzing the DEHP released, and it is more practical to use the alternative extraction solution instead of human blood. While extraction with an alternative solvent is a useful and sensitive screen, it is not likely to be representative of actual bio-availability of DEHP when used clinically in contact with whole blood. If an extraction using ethanol/water detects no or safe levels of DEHP, then this result is strong evidence that there is no need to proceed further. However if extraction with ethanol/water produces levels of DEHP which approach or exceed the TI, this does not necessarily translate to hazardous exposure to DEHP in clinical use. Instead, in such circumstances an appropriate next step would be to investigate actual availability in further studies with whole blood.
